# Carotid Intima Media Thickness Is Independently Associated with Male Gender, Middle Age, and IGF-1 in Metabolically Healthy Obese Individuals

**DOI:** 10.1155/2014/545804

**Published:** 2014-01-28

**Authors:** Hala Abd El-Hafez, Mohamed M. Elrakhawy, Azza A. El-Baiomy, Mervat M. El-Eshmawy

**Affiliations:** ^1^Internal Medicine Department, Mansoura Specialized Medical Hospital, Faculty of Medicine, Mansoura University, Mansoura 35516, Egypt; ^2^Radiology Department, Mansoura University Hospital, Faculty of Medicine, Mansoura University, Egypt; ^3^Clinical Pathology Department, Mansoura University Hospital, Faculty of Medicine, Mansoura University, Egypt

## Abstract

*Background/Aims*. The effect of benign obesity on subclinical cardiovascular disease is still questionable. The purpose of this study was to assess carotid intima media thickness (CIMT), as a marker of subclinical atherosclerosis, and to evaluate its relation to age, sex, and IGF-1 in metabolically healthy obese (MHO) subjects. *Methods*. A total of 75 MHO subjects and 80 age, and sex matched healthy nonobese control subjects were included in the study. Body mass index (BMI), waist circumference (WC), blood pressure, fasting plasma glucose, fasting insulin, HOMA-IR, lipid profile, insulin like growth factor-1 (IGF-1), and CIMT were assessed in all subjects. *Results*. MHO subjects had significantly higher CIMT and lower IGF-1 than healthy nonobese controls. Mean CIMT was significantly higher in MHO men age subgroup range from 30 to 50 years than in their age range matched (premenopausal) MHO women subgroup. In MHO subjects, CIMT was positively correlated with age, BMI, WC, SBP, HOMA-IR, TG, and LDL-C, and negatively correlated with IGF-1. Regression analysis revealed that middle age, male sex and IGF-1 remained independently associated with CIMT in MHO subjects. *Conclusion*. CIMT is elevated and IGF-1 is reduced in MHO subjects, and CIMT is independently associated with male gender, middle age, and IGF-1. Definition of healthy obesity may be broadened to include IMT measurement.

## 1. Introduction

Metabolically healthy obese (MHO) term refers to obese individuals who are relatively insulin sensitive and normotensive and have favourable glucose and lipid profile inspite of high levels of obesity [1, 2]. Evidence suggests that MHO individuals as based on BMI criteria may account for as much as 20–30% of obese population [3, 4]. MHO subset of individuals is relatively protected from obesity related cardio-metabolic disturbances that increase cardiovascular disease (CVD) risk in metabolically abnormal obese [5]. However, data concerning the exact risk of CVD in MHO as compared to healthy normal weight individuals is limited, and most data are confined to women at narrow age range.

Carotid artery intima media thickness (CIMT) is a non-invasive surrogate marker of subclinical atherosclerosis and an indicator of CVD risk [6, 7]. In addition, IMT is associated with age, male gender, obesity, and traditional risk factors [8–11].

Beside traditional CVD risk factors, obesity is associated with changes in insulin like growth factor-1 (IGF-1) [12, 13] which may be linked to atherosclerosis. Previous studies concerning the association between IGF-1 and atherosclerosis have led to conflicting results; some authors found positive association between IGF-1 and IMT in men [14] and in both men and women [15], while others found inverse association between IGF-1 and carotid IMT in healthy women [16]. Another study in middle aged subjects showed that IMT and IGF-1 are positively associated in women and inversely in men [17].

Greater subclinical CVD burden in metabolically benign obese individuals compared to metabolically healthy lean women was recently reported [18]. Further studying of subclinical CVD profile in MHO men and women and investigation of its correlates may help in proper management of MHO individuals. The aim of the present study was to assess carotid intima media thickness (CIMT) as a marker of subclinical atherosclerosis in metabolically healthy obese (MHO) subjects as compared to healthy nonobese subjects. Also we evaluated the relation of CIMT with age, sex, and IGF-1 in MHO subjects.

## 2. Subjects and Methods

The study comprised 75 MHO subjects (37 men and 38 women, mean age 48.85 ± 11.59 years) and 80 age, and sex, matched healthy nonobese (BMI 20–24.9 kg/m²) control subjects (Table 1). Obesity was defined as BMI of ≥30 kg/m^2^. Healthy obese adults were included in the study, and they were recruited from obesity Clinic of Specialized Medical Hospital, Mansoura University, Egypt. All subjects signed an informed consent to be included in our study. This study was approved by the local ethical committee.

All participants were healthy without known acute or chronic diseases. Exclusion criteria included smoking, alcohol consumption, cardiovascular, inflammatory or metabolic diseases, known or suspected pituitary, thyroid, adrenal or gonadal dysfunction, pregnancy, abnormal liver function tests, and medications (antihypertensive, lipid lowering, hypoglycaemic agents, growth hormone, anabolic steroids, glucocorticoids, hormone replacement, and hormonal contraception).

All participants were subjected to thorough medical history and clinical examination. Anthropometric measurements including height, weight, body mass index (BMI), and waist circumference (WC) were obtained using standardized techniques; height was measured to the nearest 0.5 cm, body weight was measured to the nearest 1.0 kg using mechanical weight scale (in the fasting state in the morning while wearing light clothes and no shoes). BMI was calculated as weight/height^2^ (kg/m^2^), and WC was measured at the highest point of the iliac crest at the end of normal expiration. Blood pressure (BP) was measured in the sitting position, with a mercury sphygmomanometer after a 10-minute rest. Measures were taken from the upper arm with an appropriately sized cuff. The systolic and diastolic blood pressure were read to the nearest 2 mmHg and recorded at the appearance (phase I) and disappearance (phase V) of Korotkoff's sounds respectively. The lowest of three consecutive readings was recorded.

Metabolically healthy state, defined as having 0 to 1 out of six cardiometabolic abnormalities including (1) BP ≥130/85, (2) fasting triglyceride level ≥150 mg/dL, (3) HDL-C <40 mg/dL in men and <50 mg/dL in women, (4) fasting glucose ≥100 mg/dL (ATP-III cut-off values) [19], (5) HOMA-IR >2.7, (6) high sensitive C-reactive protein (hs-CRP) >3 mg/L (Cutoff values for HOMA-IR and hs-CRP were obtained from Karelis and Rabasa-lhoret) [20].

### 2.1. Laboratory Assay

Fasting blood sample (10 mL) was drawn from cubital vein of each participant in the sitting position between 8.00 and 10.00 a.m., after 12 hours of fasting. Each blood sample was divided into two tubes, one of them containing EDTA for preparation of plasma and the other for preparation of serum. Plasma glucose and serum lipids were assayed immediately, and the remaining of the samples were kept in aliquots at −70°C till the time of the rest of assays.

Fasting plasma glucose (FPG) was estimated using commercially available kit supplied by Human (Germany). Fasting serum insulin was assayed by a solid-phase, enzyme-labeled chemiluminescent immunometric assay using immulite analyzer supplied by Siemens (USA). Homeostasis model assessment of insulin resistance (HOMA-IR) index was calculated according to the formula: HOMA-IR = fasting insulin (*μ*U/mL) × fasting glucose (mmol/L)/22.5 [21]. Total cholesterol (TC), triglyceride (TG), and high density lipoprotein cholesterol (HDL-C) were assayed by commercially available kits, Cobas (Integra-400) supplied by Roche Diagnostic, Germany. Low density lipoprotein cholesterol (LDL-C) was calculated according to Friedewald et al. [22]. High sensitivity CRP (hs-CRP) was estimated using an immunoenzymometric assay supplied by Monobind Inc., Lake Forest, CA 92630, USA. Serum IGF-1 was estimated by immunoenzymometric assay supplied by Roy Bio (USA) with intraassay CV <10%, inter-assay <12%, and no cross-reactivity with other cytokines.

### 2.2. Measurement of Carotid Intima Media Thickness

**Figure 1:**
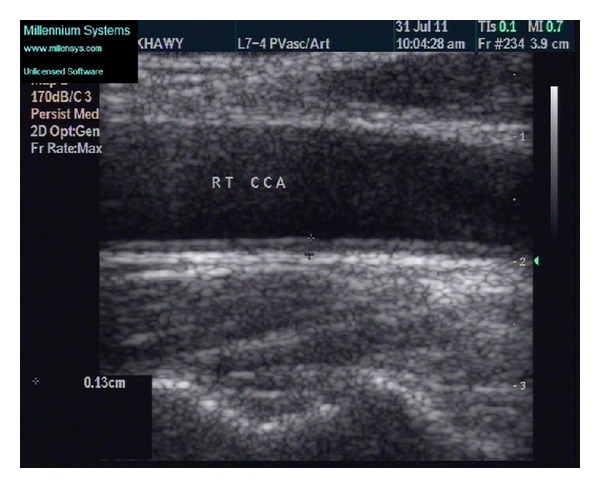
B mode longitudinal scan of the right common carotid artery in metabolically healthy obese individual showing clear lumen and carotid intima media thickness measuring 1.3 mm.

Carotid IMT was measured by B mode ultrasound using high frequency transducers 4–7 and 5–12 MHz; all subjects were examined with ATL 5000, USA, and Philips 11xe, The Netherlands. Subjects lie supine with slight head tilt to the contralateral of the examined side, with elevation of the shoulder to stretch the neck in subjects with short neck. Cases were examined by one examiner who was unaware of participant's clinical characteristics. Common carotid artery (CCA) was scanned bilaterally (in the longitudinal and transverse views) 1 cm before carotid bulb over a length of 1 cm at the far wall of both CCAs. IMT thickness was measured at plaque free segments only as the distance between the leading edge of the lumen-intima interface and media-adventitia interface. Two measurements were taken from each side and averaged; then, the mean carotid IMT was calculated as the average of measurements obtained from both CCAs (Figure 1).

### 2.3. Statistical Analysis

Data entry and analysis were performed using SPSS statistical package version 15. The quantitative data were expressed as mean ± SD. Student's *t*-test was conducted to compare the mean of continuous variables in two groups. Pearson's correlation coefficient was used to test correlations between variables. Linear regression analysis was done to identify predictors of CIMT. *P* value <0.05 was considered as significant at a 95% confidence interval.

## 3. Results

Baseline characteristics of the MHO subjects and nonobese healthy controls are shown in Table 1. MHO subjects had significantly higher SBP, fasting insulin, HOMA-IR, TG, LDL-C, CRP, and CIMT (0.78 ± 0.15 versus 0.67 ± 0.10) and significantly lower HDL-C and IGF-1 (203.7 ± 40.3 versus 229.4 ± 57.5) than those of healthy nonobese subjects.

MHO participants were divided into 2 groups according to sex. Compared to MHO women, MHO men showed significantly higher LDL-C and lower HDL-C. No significant difference was observed between MHO men and women regarding fasting insulin, HOMA-IR, TG, hs-CRP, and IGF-1 (Table 2).

**Figure 2:**
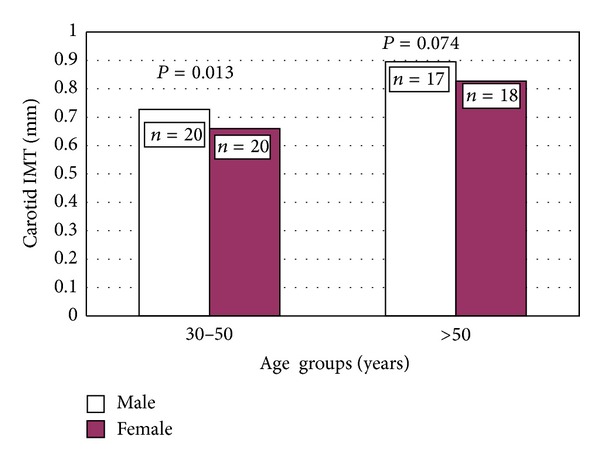
Carotid IMT in MHO women subgroups: from 30 to 50 (premenopausal) and above 50 years as compared to their age range matched MHO men subgroups.

CIMT was significantly higher in MHO men than in MHO women (0.82 ± 0.16 versus 0.74 ± 0.14) (Table 2). MHO women were divided into 2 age subgroups with respect to menopausal state: from 30 to 50 year (premenopausal) and above 50 years (after menopause), and they were compared to their age range matched subgroups of MHO men; CIMT was significantly higher in MHO men age subgroup ranged from 30 to 50 years than that in their age ranging matched pre-menopausal MHO women. However, the difference in CIMT between MHO men and women age subgroups above 50 years was insignificant (Figure 2).

The correlation between CIMT and other parameters in MHO subjects is shown in Table 3. CIMT was positively correlated with age, BMI, WC, SBP, HOMA-IR, TG, and LDL-C and negatively correlated with IGF-1.

Regression analysis was done to identify predictors of CIMT in MHO (Table 4). Age, sex, SBP, BMI, WC, HOMA-IR, TG, LDL-C, and IGF-1 were entered in the regression model. Only middle age, male gender, WC, and IGF-1 remained independently and significantly associated with CIMT in MHO subjects.

## 4. Discussion

The exact effect of metabolic healthy obesity on subclinical cardiovascular disease particularly in relation to age, sex,and different risk factors is not clearly defined. In the current study, we assessed CIMT and its relation to age, sex and IGF-1 in MHO. The main finding was elevated CIMT and reduced IGF-1 in MHO subjects; furthermore, IMT in MHO subjects was independently associated with male gender, middle age, and IGF-1.

Our results are in agreement with previous findings of higher CIMT in metabolically benign overweight/obese women compared to normal weight women [18]. In addition Ärnlöv et al. [23] confirmed that the risk of CVD and mortality is elevated in MHO individuals compared to metabolically healthy and lean individuals. In contrast, Park et al. [24] found similar CIMT in healthy obese and healthy normal weight individuals.

Despite relatively favourable cardiometabolic profile in MHO as compared to metabolically abnormal one [1, 2], MHO individuals appear to have greater subclinical CVD than normal healthy individuals. These data go in line with our findings of less favourable metabolic profile in MHO as indicated by significantly higher fasting insulin, HOMA-IR, TG, and LDL-C, and significantly lower HDL-C. Obesity itself may contribute independently to carotid structure and function abnormalities and early atherosclerotic changes in obese are only partially explained by traditional CV risk factors [25]. Obesity may modulate CCA diameter and may induce adaptive changes in carotid wall thickness independent of metabolic and atherosclerotic factors [26]. In addition to the increase in BP, obesity is associated with an elevation in total blood volume, basic cardiac output, and heart rate, which can induce new intima proliferation through changes in arterial wall stress [27].

In addition, we found significant correlations between CIMT and BMI, WC, SBP, TG, LDL-C, and HOMA-IR in MHO subjects. However, among all previous cardio-metabolic variables, only WC was a significant predictor of CIMT. Lakka el al. [28] demonstrated that abdominal obesity can be associated with progressive increase in CIMT independently of general obesity and other risk factors. In healthy individuals, WC as a measure of abdominal obesity correlates better than BMI with subclinical atherosclerosis evaluated by CIMT [29]. De Michele et al. [30] also found a significant association between WHR and carotid wall thickening independent of fasting insulin concentration. We suggest that the presence of visceral obesity is more important than insulin resistance in healthy obese and the effect of insulin resistance may be confounded by WC, and some measured (IGF-1) or unmeasured factors.

Our findings suggest that abdominal obesity in MHO individuals may exert an independent effect on atherosclerosis and it may mediate the effect of obesity on early atherosclerosis so, reduction of WC should be a target not only in metabolically abnormal obese subjects but also in MHO subjects.

Less favourable inflammatory profile indicated by significantly higher hs-CRP levels was detected in MHO subjects. We found insignificant relation between hs-CRP and CIMT. In agreement, Sinning et al. [8] found no independent association between CIMT and CRP. It is suggested that CRP is more predictive for cardiovascular prognosis in subjects of established carotid atherosclerosis [31].

In addition, age was the most significant predictor of CIMT among MHO subjects; this is consistent with previous studies reporting an association between IMT and age in both genders [8, 9]. Aging induces intrinsic changes in the arterial wall including progressive increase in intimal thickness [32], and this constant increase in IMT over age is an effect present in both male and female [33].

In accordance with a previously reported gender difference in IMT [8], we found that MHO men had significantly higher CIMT than MHO women, and the male gender remained independently associated with CIMT; this can be explained in part by a small increase in LDL-C and decrease in HDL-C in our MHO men as compared to MHO women. Furthermore, the difference in CIMT between MHO men and women in the current study seems to be influenced by menopausal status because it was more significant between middle age, than older age, subgroups. Tan et al. [9] reported that traditional risk factors explain only a small amount of gender variance in IMT, supporting the hypothesis that other sex hormone-related, behavioural, or genetic factors may play a role in the gender differences.

IGF-1 is a nontraditional factor which has been linked to atherosclerosis and obesity [12, 34]. We observed that MHO subjects had lower IGF-1 levels than healthy nonobese subjects which goes in line with the negative association between IGF-1 and BMI found by others [12, 13]. The relation between obesity and IGF-1 is complex; it is hypothesized that free IGF-1 increases with body weight until it reaches a level that triggers a negative feedback that would suppress growth hormone secretion (GH) which in turn would result in decreased IGF-1 production in the liver [35].

IGF-1 was a significant negative predictor of CIMT in our MHO subjects. These results are parallel to those of Marini et al., who suggested that low IGF-1 levels could contribute to early atherosclerosis in MHO women compared with non-obese women [16]. On the other hand, increased risk of atherosclerosis among individuals with low IGF-1 was previously reported [34]. In contrast, some found positive association between IGF-1 and IMT in men [14] and in both men and women [15]. Another study in middle aged subjects showed that IMT and IGF-1 are positively associated in women and inversely in men [17]. This seems to be a conflict, however the relation between IGF-1 system and CVD is complex, and there are many positive and negative associations. Both disorders of GH/IGF-1 axis activity including GH deficiency characterized by low serum IGF-1 and acromegaly accompanied by IGF-1 excess are associated with increased intima thickness [36, 37], So, a probably U-shaped association between the GH/IGF-1 axis activity and atherosclerosis was suggested though direct mechanisms of such a relation need to be elucidated [14].

Some speculate that IGF-1 is a proatherogenic by exerting an antiapoptotic effect on vascular smooth muscles and endothelial cells, and by being a promigratory factor [38], however, a detailed atheroprotective effect of IGF-1 has been fully reviewed [39]. Atherosclerosis is a chronic inflammatory disease initiated by oxidative stress, so the association of low IGF-1 with early atherosclerosis in our study is consistent with its potential anti-inflammatory and antioxidant effects [40]; as well as its possible role in endothelial function through its stimulation of endothelial nitric oxide production [41, 42].

Interestingly, physical activity is an important regulator of GH/IGF system [43], also weight loss restored low levels of free IGF-1 to normal [44], this further highlights the beneficial effect of life style based weight reduction in healthy obese.

Our study has some limitations, first, the cross sectional design, second, the sample size being relatively small; however, further prospective evaluation on larger scale study may be recommended. Third, CIMT was assessed by one investigator; however, a reasonable examination protocol was done by an expert radiologist.

In conclusion, CIMT is elevated and IGF-1 is reduced in MHO subjects, and CIMT is independently associated with male gender, middle age, and IGF-1. Definition of healthy obesity may be broadened to include IMT measurement.

## Figures and Tables

**Table 1:** Characteristics of metabolically healthy obese and nonobese control subjects.

Characteristics	Metabolically healthy obese subjects (*n* = 75)	Nonobese controls (*n* = 80)	*P* value
Men/women (*n*)	37/38	40/40	0.93
Age (years)	48.8 ± 11.6	47.50 ± 12	0.48
BMI (kg/m²)	34.9 ± 3	22.7 ± 1.8	<0.001*
WC (cm)	104.6 ± 10.5	77.3 ± 6.8	<0.001*
SBP (mmHg)	120.9 ± 9.5	116.9 ± 9.8	0.01*
DBP (mmHg)	75.4 ± 10.5	74.3 ± 7.2	0.44
Fasting glucose (mg/dL)	88 ± 9.2	86.2 ± 8.2	0.18
Fasting insulin (*μ*IU/mlL)	9.3 ± 2.7	7.2 ± 2.5	<0.001*
HOMA-IR	1.9 ± 0.6	1.5 ± 0.6	<0.001*
Total cholesterol (mg/dL)	172 ± 33.1	167.9 ± 24.3	0.37
Triglycerides (mg/dL)	118.6 ± 23.7	111.9 ± 25.9	0.001*
HDL-C (mg/dL)	53.2 ± 7.3	58.6 ± 6.5	<0.001*
LDL-C (mg/dL)	96 ± 25.6	86.6 ± 24	0.02*
hs-CRP (mg/L)	1.6 ± 0.6	0.8 ± 0.3	<0.001*
IGF-1 (ng/mL)	203.7 ± 40.3	229.4 ± 57.5	0.002*
Carotid IMT (mm)	0.78 ± 0.15	0.67 ± 0.1	<0.001*

BMI: body mass index, WC: waist circumference, SBP: systolic blood pressure, DBP: diastolic blood pressure, HOMA-IR: homeostasis model assessment of insulin resistance, HDL-C: high density lipoprotein cholesterol, LDL-C: low density lipoprotein cholesterol, hs-CRP: high sensitivity C-reactive protein, IGF-1: insulin-like growth factor-1, and Carotid IMT: carotid intima media thickness. To converte lipid concentration from mg/dl into mmol/L; for total, HDL, and LDL cholesterol divide mg/dL by 38.6 and for triglycerides divide mg/dL by 88.57. Data are expressed as mean ± standard deviation; *P is significant if <0.05.

**Table 2:** Characteristics of MHO according to gender.

Characteristics	MHO men (*n* = 37)	MHO women (*n* = 38)	* P* value
Age (years)	49.32 ± 11.78	48.39 ± 11.54	0.73
WC (cm)	109.38 ± 9.35	100.03 ± 9.45	<0.001*
SBP (mmHg)	122.84 ± 9.76	119.08 ± 8.92	0.09
DBP (mmHg)	75.22 ± 13.71	75.66 ± 6.28	0.86
Fasting glucose (mg/dL)	87.62 ± 9.76	88.5 ± 8.78	0.68
Fasting insulin (µIU/mL)	9.58 ± 2.47	9.03 ± 2.92	0.38
HOMA-IR	1.97 ± 0.58	1.93 ± 0.61	0.78
Total cholesterol (mg/dL)	175.05 ± 39.34	169.13 ± 25.88	0.44
Triglycerides (mg/dL)	121.57 ± 22.48	115.84 ± 23.80	0.10
HDL-C (mg/dL)	51.35 ± 8.21	54.97 ± 5.77	0.03*
LDL-C (mg/dL)	100.92 ± 27.44	91.26 ± 23.05	0.03*
hs-CRP (mg/L)	1.50 ± 0.58	1.69 ± 0.58	0.15
IGF-1 (ng/mL)	198.16 ± 40.1	209.08 ± 40.39	0.22
Carotid IMT (mm)	0.82 ± 0.16	0.74 ± 0.14	0.02*

BMI: body mass index, WC: waist circumference, SBP: systolic blood pressure, DBP: diastolic blood pressure, HOMA-IR: homeostasis model assessment of insulin resistance, HDL-C: high density lipoprotein cholesterol, LDL-C: low density lipoprotein cholesterol, hs-CRP: high sensitivity C-reactive protein, IGF-1: insulin-like growth factor-1, and carotid IMT: carotid intima media thickness. To converte lipid concentration from mg/dL into mmol/L, for total, HDL, and LDL cholesterol divide mg/dL by 38.6 and for triglycerides divide mg/dL by 88.57. Data are expressed as mean ± standard deviation; *P is significant if <0.05.

**Table 3:** Correlation between CIMT and different parameters in MHO subjects.

Characteristics	MHO (*n* = 75)	
	*r*	*P*
Age (years)	0.57	<0.001*
BMI (kg/m²)	0.40	<0.001*
WC (cm)	0.54	<0.001*
SBP (mmHg)	0.30	0.01*
DBP (mmHg)	0.07	0.55
Fasting glucose (mg/dL)	0.17	0.14
Fasting insulin (µIU/mL)	0.27	0.02*
HOMA-IR	0.26	0.02*
Total cholesterol (mg/dL)	0.23	0.05
Triglycerides (mg/dL)	0.26	0.02*
HDL-C (mg/dL)	−0.18	0.13
LDL-C (mg/dL)	0.24	0.03*
hs-CRP (mg/L)	0.09	0.46
IGF-1 (ng/mL)	−0.47	<0.001*

CIMT: carotid intima media thickness, MHO: metabolically healthy obese, BMI: body mass index, WC: waist circumference, SBP: systolic blood pressure, DBP: diastolic blood pressure, HOMA-IR: homeostasis model assessment of insulin resistance, HDL-C: high density lipoprotein cholesterol, LDL-C: low density lipoprotein cholesterol, hs-CRP: high sensitivity C-reactive protein, and IGF-1: insulin-like growth factor-1. *P is significant if <0.05.

**Table 4:** Regression analysis of clinical and laboratory variables on CIMT among MHO subjects.

Characteristics	MHO (*n* = 75)	
	*β*	*P *
Age (years)	0.62	<0.001*
Sex (male)	0.08	0.001*
SBP (mmHg)	0.15	0.21
BMI (kg/m²)	0.12	0.46
WC (cm)	0.39	<0.001*
HOMA-IR	0.03	0.38
Triglycerides (mg/dL)	0.02	0.79
LDL-C (mg/dL)	0.02	0.63
IGF-1 (ng/mL)	−0.07	0.04*

CIMT: carotid intima media thickness, MHO: metabolically healthy obese, BMI: body mass index, WC: waist circumference, SBP: systolic blood pressure, HOMA-IR: homeostasis model assessment of insulin resistance, LDL-C: low density lipoprotein cholesterol, and IGF-1: insulin-like growth factor-1. *P is significant if <0.05.

## List of Abbreviations

MHO: Metabolically healthy obese
CVD: Cardiovascular disease
CIMT: Carotid artery intima media thickness
IGF-1: Insulin-like growth factor-1
BMI: Body mass index
WC: Waist circumference
BP: Blood pressure
FPG: Fasting plasma glucose
GH: Growth hormone
HOMA-IR: Homeostasis model assessment of insulin resistance index
HDL-C: High density lipoprotein cholesterol
LDL-C: Low density lipoprotein cholesterol
hs-CRP: High sensitive C-reactive protein
CCA: Common carotid artery.
